# Distinguishing protest responses in contingent valuation: A conceptualization of motivations and attitudes behind them

**DOI:** 10.1371/journal.pone.0209872

**Published:** 2019-01-08

**Authors:** Ulrich J. Frey, Frauke Pirscher

**Affiliations:** 1 German Aerospace Center, Stuttgart, Germany; 2 Martin-Luther University Halle-Wittenberg, Halle (Saale), Germany; Shandong University of Science and Technology, CHINA

## Abstract

The percentage of protesters in contingent valuation surveys is substantial–about 20% across many studies. This paper seeks to clarify the motivations behind protest responses. In addition, the question whether the estimation of willingness to pay (WTP) is more biased by the *exclusion* or *inclusion* of protest bids is yet undecided. Methodological improvements are difficult for three reasons: motivations behind protest responses are largely unclear, definitions of protest differ between studies and often only participants who state a zero WTP are asked for their reasons. Our survey on farm animal welfare (n = 1335) provides detailed motivations, two definitions and includes debriefing of all participants for their WTP. We find that protest bids are not a refusal to answer, they are neither irrational nor driven by lack of understanding. Quite the contrary, a large part of participants is directly motivated by moral reasons. Furthermore, protest responses are not coupled to a zero WTP. In our sample, only 8% out of 32% protesting participants had a zero WTP. Only a small fraction of zero bids (0.4%) are true WTP-statements, i.e. respondents were satisfied with the status quo. This finding has important implications for existing WTP-estimates which might be biased. Finally, we provide detailed estimates of the WTP for animal welfare issues by including and excluding different types of protesters and outliers.

## Introduction

### Willingness to pay and protest responses

For many projects and products it is crucial to correctly estimate the willingness to pay (WTP) of individuals for a broad variety of goods, because the aim of many decisions is to maximize social welfare and the option with the highest overall utility should be chosen. The optimal choice presupposes that total utility is known. For environmental and ethical goods indirect use and non-use values (e.g. [1]), like option, bequest or existence value but not their direct use-value are constitutive. By valuing environmental and ethical goods for other reasons than individual utility allows to express people’s moral attitude towards future generations and non-human beings. However, especially for many environmental goods (e.g. the value of wetland restoration, the premium for organic food, the value of more biodiversity), it is hard to estimate the non-use value since no market for these products exist and therefore it cannot be assessed directly in monetary terms. A popular approach is to ask for stated preferences, e.g. via surveys [2].

Given technical difficulties like hypothetical or other biases in surveys using contingent valuation techniques (CV or CVM) that measure non-market resources, some authors are doubtful whether willingness to pay can be precisely measured at all [3]. In contrast, its proponents have responded to criticism with improved techniques, arguing that technical difficulties can be overcome (e.g. [4–6]).

One problem of estimating WTP, i.e. the amount of money someone is prepared to pay for a specific product or service is that WTP is often equated with the economic preferences behind it. In contrast, it has been suggested that a number of non-economic reasons influence the willingness to pay [7]. They may be moral, social or political in nature [8]. Especially controversial topics like animal welfare, i.e. the treatment an animal receives and the state it is in, in consumer goods or the provision of particular environmental goods and services are strongly intertwined with moral aspects [9–11]. Hence, for many issues, stated WTP may not reflect exclusively economic preferences [12,13].

This fact presents theoretical and methodological difficulties [7] for estimating WTP, resulting in uncertainty what exactly is measured. In particular, *protest bids* may bias the results. A protest bid is defined as not stating the true WTP value for the good in question for whatever reason [14]. Usually, all participants stating zero as willingness to pay and give a reason why they refuse to pay (i.e. do not have a genuine WTP of zero) are labeled as protesters. In a meta-analysis of 157 studies, an average 18%, but up to 59% of WTP estimates are labeled as protest bids [15]. This substantial number of zero bids, i.e. individuals showing a willingness to pay of zero may be a strong indicator for the existence of certain moral or political attitudes or other factors behind WTP.

For correctly estimating the willingness to pay for a certain non-market good, this is a critical issue. A correct classification and statistical treatment of protesters is highly relevant because estimating an unbiased WTP is important for correct policy decisions. How to deal with this substantial percentage of protesters? The difficulty consists of determining whether a willingness to pay of zero is a protest: Are respondents satisfied with the status quo (a genuine WTP of zero) or are they protest responses, i.e. bids that actually express a *positive value* for the non-market good but enter a zero amount nevertheless? Usually, surveys distinguish both groups by posing a series of debriefing questions to survey respondents with a zero bid as WTP in order to exclude them from the sample.

However, there are several difficulties with existing approaches. First, many studies ask debriefing questions only of those individuals who gave a zero bid although various studies have shown that protest beliefs are held as well by respondents who state a positive willingness to pay [16,12,17]. This is particularly problematic since only the fraction of the protest bids with zero values for WTP are removed while keeping others with a positive value [6].

Second, many studies do not differentiate more precisely between reasons of protest bids, although a variety has been identified (e.g. [18,11]). Moreover, protest bids may require different treatment because they are not equal conceptually [12,13]. Different motivations are problematic when dealing with protest responses since they may lead to a different willingness to pay.

Third, across studies, protesters are not identified with a single, but various different methodologies [19]. In addition, they are treated differently in WTP analyses by not being systematically excluded or included [17,20].

Given this situation, one contribution of this paper is to advance research by analyzing in detail the motivations behind protest responses and the conceptual link between protest and WTP. We are able to connect WTP to moral attitudes by using validated scales on moral positions. Using latent class analysis, we provide a fine-grained distinction on many attributes. Based on this we are able to classify subjects in five distinct groups. This aims to consolidate a definition of protest, hopefully leading to a more standardized definition. Finally, we address the treatment of protest responses by presenting a comparison of WTP estimates according to different inclusion criteria. Ultimately, these efforts lead to less biased WTP estimates, thus impacting on a broad range of topics.

This paper is structured as follows: in the following section, we discuss the state of the art for the treatment of protest responses before profiling motivations behind protest responses in more detail. The Methods section describes the survey design, discusses possible biases and provides two definitions of protesters. The Results part presents descriptive statistics of protesters, a latent class analysis of attitudes and WTP estimates for different groups. These results are then reviewed in the Discussion, followed by the Conclusion.

### State of the art

The following section first discusses possible bias in measuring WTP, before demonstrating that protest responses have been treated differently across studies. Finally, the motivations found so far behind protest answers are analysed.

### Measuring unbiased WTP and protest responses

The suitability of contingent valuation (CV) to estimate WTP has been called into question due to methodological problems. These include framing effects [21], differences between willingness to pay and willingness to accept [3], overestimation of WTP since questions are hypothetical [5] and poorly formed preferences [22]. In reaction to the criticism, proponents of CV provide best practices [5,23] while using increasingly more sophisticated designs [24,25].

Such criticism of many aspects of eliciting WTP (e.g. the influence of the sequence of questions, the length of vignette texts) via contingent valuation also includes the question on how to treat protesters. In addition, there is usually a group of outliers with an extremely high WTP and a group that refuses to state a WTP at all [17]. These methodological challenges raise the question whether it is possible to measure WTP in an unbiased way at all. In particular, it is unclear which answers to WTP questions constitute protest responses and which do not, since neither all zero bids are motivated by protest nor all positive WTP values are from non-protesters. It is also not clear which treatment of protest responses results in a minimal bias for WTP estimation [14].

## Different treatments of protest responses

One reason for the criticism on treatment of protest responses is that there is no standard definition of protest. Currently, protest responses are defined slightly different, which introduces an undesirable subjective bias and makes research on protest responses hardly comparable [17,12,6]. Thus, a critical issue at the heart of the problem, i.e. what constitutes a protest response, is far from solved [7]. To address this issue, this study differentiates between two frequently used definitions (see Methods for details) and profiles protesters with a method known to minimise bias (latent class analysis).

A second reason for criticism consists in different ways to treat protest responses, i.e. there is no established procedure for censoring protesters, again making results incomparable across studies [26,17]. The two most frequently used are, first, minimizing protest responses through design, e.g. through pre-tests or entreaties [26], and second, the removal of all protest bids [20]. In contrast, using imputed values for protesters [27] or treating protest responses as legitimate WTP are seen as problematic since both methods may bias WTP significantly.

However, the very removal or exclusion of protest bids to arrive at an unbiased WTP [19,28] may lead to a biased WTP. This bias has been demonstrated by other studies comparing WTP with and without protesters [27,29]. The latter results show that protest responses change WTP estimates considerably. However, it is yet inconclusive in these studies whether exclusion leads to a higher or lower WTP: some find major positive effects of including protest bids (e.g. an increase of 46% in WTP, [30]); others find a significant negative relationship between protest bids and WTP [17]. This points to a context-dependency, which may be even more pronounced in highly emotional issues like animal welfare.

Excluding a substantial percentage of a sample (11%-59%, which is the range of protesters found in a meta-analysis, see [15], risks to miss relevant motivations behind those answers. It has also been suggested that important ethical issues are connected to protests as well [8]. Therefore, protest bids are part and parcel of WTP, since they are related to other answers in each respective survey (for specific relationships see [30,17]. They are also not independent of the survey format itself [31]. Thus, it can hardly be justified to delete parts of the survey if there is a shared but perhaps unknown (moral) attitude behind WTP decisions [12].

More generally, from a statistical point of view, such censoring is undesirable. If a majority of studies excludes protest bids, findings cannot be generalized [7], because removing protest bids destroys systematic relationships, resulting in sample selection bias. This is one of the reasons we employ a latent class analysis to identify groups behind survey answers.

As a conclusion, there is no established procedure on how to classify protest responses in regard to WTP [17] and deleting them is no option. We will show (see Results) that it is important for an unbiased estimation of WTP to differentiate protest responses according to their motivation. It is also important to distinguish protests from refusals to answer, irrational responses and from individuals who did not understand the question.

These different definitions and inconsistent treatments of protest responses suggest analyzing in more detail what reasons and motivations are behind protest responses. This might lead to an adapted and differentiated treatment which in turn could help to minimize bias in estimating WTP.

There are several reasons that have been identified in the literature why subjects protest: a general objection to the survey, the payment vehicle (e.g. fees, taxes or a price premium) or the manner of the questions posed; a conviction that either the government or others should pay for the good; the fact that more information is needed to answer; that it is unfair to ask for money for the good; that one should not have to pay for the good as it constitutes a basic right; strategic behavior; refusal to play the game or that no monetary value can be placed upon the good [12,20,11,32]. In contrast, answers that serve as indication for a true WTP of zero are, for example, “I cannot afford it”, “The good in question is not important”, “I do not care about this issue” [30].

## Profiling protest responses: Motivations and demographics

Generally, heterogeneity in protest response patterns has been linked to differences, for example, in race, gender, culture and country [33]. Besides general objections for protesting, protest responses are also associated with certain kinds of *motivations and attitudes*. More specifically, some studies analyze protest responses and the motivations behind the willingness to pay for environmental concern [17,30,12]. However, it is yet unclear whether protesting individuals constitute a group with a particular profile in demographics or moral values. Therefore, it seems especially relevant to profile these groups in terms of demographics, moral values and their specific motivations for issues like animal welfare, where changes are increasingly demanded by society. Animal welfare issues are particularly interesting since these moral decisions are tightly coupled to a good that is well-known and regularly consumed on markets–which should reduce bias in WTP estimation.

Existing studies associate protest behavior with the following variables:

- lower environmental concern and lower knowledge of the good in question [6,17,30,31,12]
- older, less educated and low income participants [30,19,31,34]
- no gender differences [31,20]; but see [34] who find a higher probability of being male and a protester)
- fairness and equity concerns or the expression of a certain moral attitude [12,18,8]
- various survey characteristics, e.g. the payment vehicle [15]

To sum up, protesters do not seem to constitute a random group of the sample surveyed. This argument is supported by research that finds that classifying protest bids does not result in a coherent sub-sample [35]. For example, contrary to expectations, there are some respondents who pay, i.e. have a positive WTP but who are identifiable as protesters according to the usual classification (a WTP of zero and a reason classified as protest, [14]. Hence, it seems difficult to reliably separate the protest group from the rest of the sample [27] because often non-zero bids are not asked debriefing questions. Thus the current debate on protest bids is characterized by an information deficit and an associated definition problem. This article tries to disentangle these issues by debriefing all participants and by estimating WTP based on different definitions of protest responses.

Three research questions are addressed. First, which motivations and moral attitudes are behind protest responses? Second, do protest responses constitute distinguishable groups, differing e.g. in demographics and moral values? Third, does WTP differ significantly with protest bids, zero bids, outliers included or excluded?

These research questions translate into the following hypotheses:

Hypothesis 1: Protest bids do not reflect irrationality, lack of understanding or a refusal to answer.

Some studies discuss whether and how protest responses might be a sign of irrational behaviour [6,36]. Thus, it has to be determined whether this is the case and how large this group might be. Completely irrational answers may in fact be one of the a priori valid reasons to remove participants from the sample since these are certain to bias WTP.

Hypothesis 2: There are moral attitudes behind protest bids. Certain moral attitudes, like more environmental concern lead to less protest bids.

The classical economic assumptions for WTP is the aim to maximize individual utility. Since the WTP for non-use values of environmental and ethical goods is morally driven, it is worth analyzing whether different moral attitudes, especially for debated issues like animal welfare, may be directly connected to protest answers [8,9]. One goal of this article is to explore possible connections further to contribute to the question whether WTP analysis is able to capture moral attitudes besides those that are based on anthropocentric utility.

Hypothesis 3: Protest responses constitute distinguishable groups, differing e.g. in demographics and moral value. They are not necessarily associated with a zero WTP.

In order to improve our knowledge about who is likely to give a protest response, we apply latent class analysis to be able to identify clusters of groups in a more nuanced way.

Hypothesis 4: WTP differs significantly with protest bids, zero bids or outliers excluded.

Since various studies treat zero bids and outliers differently (see discussion above), this hypothesis tries to shed light on these issues by directly comparing the effect on WTP by excluding different groups.

Understanding motivations behind WTP for non-market goods and identifying groups of protesters might improve the conceptualization of protest and help to address missing comparability across studies. Exploring reasons behind protest bids may also add to the debate whether contingent valuation is more about economic preferences or more about expressing certain attitudes [12,13]. If understood as an expression of attitudes, WTP should be understood as a non-economic statement of moral, social and political points of view.

## Methods

There were no known risks associated with this research study, participants of the survey and the interviews were not a vulnerable group of people, and complete confidentiality was guaranteed. Questionnaires were completed anonymously: No information was collected that could be used to identify participants. Participants knew that they were involved in a scientific experiment, and were asked for their consent to use the data. None of the participants expressed discomfort or asked to withdraw their data from the study.

### Survey

We conducted a survey on farm animal welfare and WTP both online and via paper form questionnaires from 15^th^ of December 2015 to 19^th^ of February 2016 in Germany. Three rounds of pre-tests with about ten persons with and without scientific background reduced possible ambiguities in the questions. Pre-tests were repeated during two months with the same persons until there were no more misunderstandings. The online and paper form versions were identical. The paper form version–where subjects had to fill out a printed form–was conducted in several public locations (e.g. city registry office) and made up only 4.2% (69 participants) of the total sample. The majority of respondents were contacted by a university mailing list (n = around 18,000) in order to reach a sufficient number of participants. Out of 2672 responding participants (return rate ~ 15%), 1660 (62.1%) completed the survey. Since this is a satisfactory response rate for surveys of this kind, we did not investigate any potential non-responsive bias further.

To mitigate possible bias, the topic animal welfare was neither mentioned in the title nor the introduction. A randomizing mechanism in the survey software Limesurvey 2.06 (https://www.limesurvey.org/en/) assigned participants to two treatments. The paper form version was randomized as well. The only difference between the two treatments A and B was the visibility of the justification of WTP answers. In treatment A, all justification options were visible from the start. In version B, these options only popped up if either a WTP of zero or a value of > 40% of the typical product price mentioned in the description was entered. This does not mean that those who did not see the justifications (answer options) were not affected by their attitudes or personal traits [37]. However, there was no treatment effect (A or B version) on any of the four dependent variables, i.e. the four WTP questions in both the questionnaire and online versions (two-sided t-test, n.s.). Therefore, the data was pooled for all further analyses. The analyses were conducted with R 3.2.3 [38].

The survey consisted of five sections in this order: demographics, WTP questions on aspects of animal welfare, questions on the environmental attitude, including the scale for General Awareness of Consequences (GAC, see [39,40] and scales measuring moral values, e.g. altruistic tendencies and environmental apathy [41]. All questions making up the scales can be found in the Supporting Information (S2 Text). Scales were not normalized in order to enable comparisons with other articles using the same scales. The fourth section inquired about other aspects of animal welfare. The final section employed a validated scale on deontological and utilitarian values [42]. Deontological positions hold that moral actions are in themselves right or wrong, regardless of their actual outcome. In contrast, utilitarian positions define the moral quality of an action precisely according to its consequences.

Taken together, there were 37 questions that took around 20–30 minutes to answer. Most questions used a 5-point-Likert-scale format using only two ranges: "very important" to "not at all important" or "strongly agree" to "strongly disagree".

We designed a decision framework of a shopping situation for well-known animal products. We also provided the typical price of these products. Through an open question we asked for the WTP as price premium for the animal product if the animal received some clearly defined animal welfare improvements before. We chose different animals and different kind of animal welfare improvements (staying alive, reduced physical pain, more space).

The complete text of all four WTP questions for animal welfare can be found in the Supporting Information (S1 Text). In short, the first question asks participants about their WTP for eggs if male chickens are not killed. The second question is about WTP for more space for pigs, the third one asks about WTP for pain medication for the castration of piglets and the fourth one about WTP for more space for chickens. Here is the full text for the first two questions:

1. To produce eggs only female animals are needed. Male chicks are therefore killed on their first day for economic reasons. At the moment six eggs produced on deep litter farming cost 1.32 Euro. How much more would you pay for six eggs if male chicks could be raised as broilers (in Eurocent)?
2. In accordance with the German animal protection law the minimum space for fattening pigs isfattened pigs are allowed to have, depending on their weight, between 0.5 and 1 m^2^ space. Animal rights activists demand more space. At the moment, a chop of meat from pigs (1 kg) costs around 4.95 Euro. How much more would you pay for 1 kg of pork if pigs were accorded 1 m^2^ more space (in Eurocent)?

A few typing errors were corrected, if they were obvious. For example, the year of birth 19993 was corrected to 1993. Scale items were aligned in one direction and added to obtain an index value. Twenty participants (1.2%) entered extremely high values for one or more WTP questions like '9999999'. If these participants also explained these values by commenting in the sense of „This is an expression of my high WTP“, and did not articulate any protest in the text fields these values were adjusted to the highest values occurring in the survey (30 Euro for both chicken-related WTP and 100 Euro for both pig-related WTP). Therefore, these few demonstratively high but misleading values remain outliers but do not introduce further bias. Instead of simply excluding them, this unusual step ensures that the analysis which is concerned about outliers, does not censor them. The Supporting Information contains all relevant questions (S1 Text), scales (S2 Text) and their factor loadings (S2 Text, S1 and S2 Tables).

All vegetarians and vegans (n = 325) are excluded from further analysis based on their answer on the respective question in the survey since we are asking about WTP for a good that they do not consume. The remaining 1335 participants were used for further analysis.

### Possible bias

In every survey, the possibility of biasing participants through survey design exists. The next section discusses some possible biases. Great care was taken that the vignettes (see S1 Text) were neutrally phrased and were concise but presenting the context sufficiently. Several rounds of pre-tests minimized ambiguities. We are aware that people's willingness to pay is often overestimated through the hypothetical nature of the questions leading to considerable upward-bias [3,5]. However, in our case the setting in the description (a decision to buy in a supermarket with a typical price for the product given) is very familiar and should mitigate bias (see [43]. From this setting, the payment vehicle is obvious to participants–an individual price premium for each product (eggs or steak) in a familiar market situation. Participants had to state how much exactly they would be willing to pay more for a certain product, if certain ethical standards were increased.

Another issue is that of respondents giving strategic answers, i.e. stating a high WTP, because participants “… believe that their responses might influence the actions taken by businesses or governments (hereafter, agency)”[44], p. 182). It was made clear to respondents that the survey was from a university and its goal was described in the introduction as purely scientific. Therefore, it is hard to see how strategic answers could play a major role. Besides, there is no link from a university survey to buying private goods in a supermarket and there was no mentioning of any practical consequences nor agenda. Moreover, practically all surveys we all are confronted with are for purely information-gathering purpose. Hence, we conclude that the usual expectation of a survey-participant is exactly this–disclosing information about a topic, but not stating prices that will be implemented based on his/her numbers given in a survey.

Ordering bias in the questions may exist, but is deemed unproblematic, since the content of the four WTP questions is sufficiently different. With these four particular questions it is hard to see what the ordering effect could be. In addition, this problem is handled by latent class analysis [33], see also below). The 5-point Likert-scales were presented in a visually intuitive and unvaried way so that all five answer possibilities were present at all times. To avoid bias through the type of questions asked about environmental and moral issues, these questions were placed after WTP questions.

As elicitation format we chose four open-ended questions about WTP for animal welfare. We chose this and not a payment card format, because the open-ended nature of questions is more suited to identify protesters among respondents who refuse to pay [45,19]. Other formats introduce their own respective biases. For example, surveys with discrete choice format seem to result in a higher WTP [46,29].

Yet, the text (vignette) describing the questions resembles payment card format, since we provided the typical price of the product in question to avoid uninformed answers [47]. The key to avoid bias is not so much the format per se, but the familiarity of respondents with the product and situation [43], which in our case was very high, thus minimizing possible bias. This can be backed up by the fact that no participant mentioned unfamiliarity with any part of the survey in the debriefing answers or that it was inappropriate per se. By offering welfare improvements for different animals we controlled for biases that may be caused by sympathy or antipathy for a certain animal species.

Finally, non-response bias might be an issue. The response rate of 15% might be low for a survey distributed by letter–but for an e-mail based survey this is satisfactory. A meta-study finds response rates as low as 5% (mean: 33%, cf. [48]). We try to estimate whether non-response bias is a problem in our particular case. As suggested in the literature, this can be done by comparing the first third of respondents (via the time-stamp) with the last third. The idea is that the last third resembles the non-participants of the survey more closely. The t-test between these two groups is non-significant (p = 0.623), indicating that there is no particular reason to be worried. In addition, other studies suggest that non-response bias might not distort results at all ([49,50]).

### Latent class analysis

Latent class analysis (LCA) is a sub-method of structural equation modeling and is used to determine clusters or groups (classes) via observed variables [51]. Classes are discrete and not directly measurable. Each variable has a certain probability of belonging to a class. This allows constructing meaningful classes from the variables.

One main reason to use LCA was to avoid bias in the grouping procedure of protest responses according to attitudes. Latent class models have been successfully employed for the identification and classification of protesters [16,33]. Since class membership is calculated according to the statistical independence of variables, selection bias is eliminated. As a result, the differences in attitudes behind stated WTP that are not directly observable (latent) can be modeled and groups can be distinguished according to the probability of belonging to a particular class. Group sizes can also be calculated, which is relevant for policy decision makers [16].

The variables used for inclusion in the attitudinal model are: gender, income, age education, number of children, role of animal welfare in legislation, suffering of animals and humans in comparison, absolute rights for animals and humans, deontological/utilitarian attitude 1–3, shopping behavior, knowledge about animal welfare, member of an animal welfare organization, vegetarian, altruism index, apathy index, apathy towards environmental issues, meat consumption, animal welfare as moral issue, animal welfare as individual responsibility, protest definition 1, protest definition 2 and WTP for each of WTP questions.

The composition of the indices including factor loadings can be found in the Supporting Information (S2 Text and S1 and S2 Tables). Income, age and consumption of meat were recoded into ordinal categories. WTP was recoded into a WTP of 0, positive values, outliers (see above) and no values given, i.e. four classes.

Models were estimated with R 3.2.3, package poLCA [38]. Six classes (from n = 1, which corresponds to a loglinear independence model to 6 latent classes, more classes were not considered to be meaningful) were calculated, with a maximum of 3000 iterations per model and 30 instead of 1 repetition and different seeds to maximize the chance to reach the global maximum of the log-likelihood function. All six models converge, which speaks for the quality of the indicators [51].

### Definition of protest responses

It is one aim of this paper to clarify what constitutes a protest response (Table 1). For this reason, we use two different definitions of protests that are compared throughout the results section. The first definition (subsequently referred to as definition 1 or “protests with zero”) includes those participants that *state a WTP of zero in combination with a protest answer*. A protest answer is one of the following [7,30,6,12,17]:

**Table 1:** Answer options, percentages for answers given and coding as protest response for reasons for WTP questions.

Answer option for WTP	Answer	Mean Percentage (all answers)	Mean Percentage (protest bids, definition 1)	Mean Percentage (protest bids, definition 2)	Protest Yes / No
**1**	I already pay enough for food.	1.09	1.17	3.69	No
**2**	It is unfair to ask me to pay.	0.22	0.54	1.33	Yes
**3**	Animal welfare is a moral question and cannot be regulated with money.	8.05	20.80	15.48	Yes
**4**	The question is too difficult / too complicated / I need more information for a decision.	3.87	10.07	14.65	Yes
**5**	Animal welfare is no goal for me.	1.73	0.33	0	No
**6**	Number just invented / guessed / no special reason.	3.83	4.12	2.62	No
**7**	Animal welfare problems cannot be solved by individuals. Therefore the government should deal with it (e.g. via taxes or fees), not me.	5.20	13.45	14.26	Yes
**8**	I can expect animal welfare and should not pay for it.	1.04	2.73	6.10	Yes
**9**	I already spend much for animal welfare initiatives.	0.27	0	0	No
**10**	Animal welfare is really important. I want to express this with my willingness to pay. I want to contribute in a fair manner compared to others.	57.30	25.64	9.91	No
**11**	Other: free text entry	17.40	21.15	31.98	Depending on entry (if like 2, 3, 4, 7, 8 then Yes, otherwise No)

This classification of protest results in 8.7% of protest responses. Around 1.8% of this group state as reason "Animal welfare is no goal for me.", thus indicating that their zero bid is an expression of a true WTP. Therefore, 98.2% of zero bids do not express a true WTP, i.e. a matching statement of the irrelevance of animal welfare to a zero bid.

A second definition (referred to as definition 2 or “protest only”) does not link protest responses to a WTP of zero [12,16,13]. Therefore, a protest response is anyone who gave one of the listed reasons above (Table 1, column 6). Thus, it was not necessary to have a zero WTP to be classified as a protest response after this definition. Accordingly, a much higher proportion, 32.3%, is classified as protest bids.

For some analyses, we distinguish outliers as a third group. Outliers are unusually high bids, defined by a lower boundary of the interquartile range for each WTP question * 1.5. This results in a proportion of 17% / 8% / 12% / 10% (n = 232 / 110 / 159 / 139) of outliers in the sample for WTP for pay to prevent killing male chickens / more space for pigs / pain medication for castrating pigs and more space for chickens respectively. A percentage of 4.8% (n = 64) has high bids for all four WTP questions. Outliers are thus distinguished from the other three groups identified in this article–protest bids with zero, protest only and rest of the sample. A check of the text comments of outliers confirms that no one did not take the survey seriously or had a lack of incentive compatibility (see data repository for all answers).

It is worth noting that only 3.3% found the WTP questions too difficult to understand or would need more information to answer them. This confirms that the three rounds of pre-tests cleared up the majority of possible ambiguities. In addition, the comments confirm that most needed more information about this issue, but could understand the question without problems.

## Results

We begin with the descriptive statistics for the survey participants and the WTP (Table 2). The 1335 respondents were, on average, 32.5 years old, 37% were male, 55% had at least a Bachelor's degree and the annual income was average (within the category between 1500 and 2500 Euros). Concerning demographic attributes, this makes our survey reasonably comparable to representative statistics (44 years old (http://www.bib-demografie.de), 49% male (http://www.bpb.de), 2716 Euro of mean annual income (http://de.statista.com). The differences are due to the sample being drawn from a university population. Results are therefore only somewhat representative for the entire population of Germany.

**Table 2:** Descriptive statistics for WTP in Euro.

	Minimum	Maximum	Mean	Median	SD
**WTP kill male chicks**	0	3.00	0.97	0.68	1.40
**WTP space for pig**	0	10.00	2.89	2.00	5.67
**WTP castration pig**	0	10.00	2.61	1.00	5.72
**WTP space chicken**	0	2.50	1.02	0.68	1.48

Recall that we were interested in differentiating protest responses more precisely, which we summed up in our first research question "What is behind protest responses?". This research question is separated into three hypotheses. We are aware that attitudinal questions are region- and issue-specific [33].

*Hypothesis 1*: *Protest bids do not reflect irrationality, lack of understanding or a refusal to answer*

The first step to understand what is behind protest responses is to analyze what they are not. For this question only version B of the survey was used to avoid any potential bias since not all participants in version A (see Methods) were presented the answer options. However, a check concerning percentages of answers given (version A compared to B with answers presented) confirms that results for both samples are practically identical.

In the overall sample, the number of individuals refusing to give an answer, i.e. leaving a blank for the four WTP questions is very small. For WTP to prevent killing male chicks it is 1.7%, for WTP to improve space conditions for pigs, it is 2.5%, for pain medication for castrating pigs 3.3% and for WTP to improve space conditions for chickens 3.1%. This is in line with missing values of all other questions, ranging from 0% missing answers to a maximum of 3.7% missing (age). Inquiring after income had 1.5% refusals to answers.

Furthermore, protest bids do not reflect irrationality. An answer is defined as irrational if a respondent checked either the answer option “WTP was guessed” (3.8%)) or the answer option “the question is too complicated, difficult or more information is needed” (3.9%). The latter points to a lack of understanding. A refusal to answer, i.e. leaving the values for WTP empty is seen for 2.7%. Hence, the overwhelming majority of protest bids are not a refusal to answer.

If the sample is restricted to protest bids (protest with zero or protest only), the percentages for irrational answers do not change, whereas the option "too complicated, I need more information" increases to 10.1% and 14.7% respectively (see Table 1).

*Hypothesis 2*: *There are moral attitudes behind protest bids. Certain moral attitudes, like more environmental concern lead to less protest bids*

One question still under debate is whether WTP has to be understood more as individual economic preferences or as moral attitudes. As shown in Table 1, there is a substantial percentage of individuals (overall: 8.1%; protest with zero: 20.8%; protest only: 15.5%) who explicitly affirm that animal welfare is a moral question that cannot be regulated with money. A mere 1.8% (0.4%; 0%) state that animal welfare is no goal for them. Notable in this respect is the "Other" category, which was used by 17% (21%; 32%). A manual analysis of these answers reveals that 12% explicitly state a moral reason while the majority of answers think that animal welfare is "really important". Thus, moral concerns are behind a number of answers.

We also measured environmental concern by the validated scale of General Awareness of Consequences (GAC, [40]; see also Methods). There is a highly significant difference for both protest groups (definition 1 and 2) and the rest of the sample (Mann-Whitney-U-Test, n = 1335, p < 0.001) concerning the GAC-score. The corresponding means of the GAC-score–ranging from 5 to 45 –are 37.2 (protest with zero) and 35.2 (protest only) respectively in contrast to 38.3 and 38.2 for the rest of the sample, indicating that the rest of the sample has a higher environmental concern than protesters.

Since two questions touch upon the consumption of pigs, religious dietary restrictions could be an issue. To check for this, we included a question whether moral questions are primarily linked to religious concerns. Only 0.3% of all participants answered affirmatively.

*Hypothesis 3*: *Protest responses constitute distinguishable groups, differing e.g. in demographics and moral value. They are not necessarily associated with a zero WTP*

By design, almost all survey participants (see Methods), not only individuals with zero bids, were asked about reasons for their willingness to pay. This enables us–in contrast to many other studies–to distinguish between all individuals that have a WTP of zero and a protest response and those with a WTP > 0 but having also stated a protest response. A first cue that differences exist is the large difference in proportion between these two groups (8.7% fulfill the definition for the first group, i.e. definition 1 and 32.3% fulfill the definition for the second one, i.e. definition 2), which supports the hypothesis that protest bids are not necessarily associated with a zero WTP.

It is important to note that only 5.2%, (n = 6) of the protest zero group (8.7%, n = 116) state as reason "Animal welfare is no goal for me.", indicating with their zero bid a non-protest WTP. The overwhelming majority of zero bids indicate therefore protest responses, making it hard to justify treating zero bids as expressing a true WTP.

To identify potential differences among protesters we first analyze their differences in demographics and morals versus the rest of the sample. Note that the data (except for age) is based on Likert-scales–we therefore cannot calculate the means, but present the direction only.

As shown in Table 3, there are significant differences for protest responses with zero bids: Overall, they are younger, more likely to be female, have less income and have fewer kin they have to care for. Protesters after definition 2 are also more likely to be female, have less income and are less convinced that animals possess an intrinsic value. These differences are all significant (Mann-Whitney-U-Test, n = 1335, p-values in Table 3, column 1). All other tests for these variables are not significant.

**Table 3:** Demographics of protest groups compared to the rest of the sample (n.s. = not significant, if n.s. cells indicating direction are left blank).

	P-values MW-test Protest with zero and normal bids	P-values MW-test Protest only and normal bids	Protest with zero	Protest only
**Age**	< 0.001	n.s.	Younger	
**Sex**	0.04	< 0.01	more female	more female
**Educational level**	n.s.	n.s.		
**Responsibility for others in household**	0.03	n.s.	less kids	
**Income**	< 0.001	< 0.01	lower	lower
**Utilitarian** **index**	n.s.	n.s.		
**Intrinsic** **Value Animals**	n.s.	< 0.01		lower

We continue our discussion of different groups and protest responses with a much finer classification of different attitudes. This is possible with latent class analysis, using 27 variables as indicators. Since more indicators generally lead to better models, this number should provide a good fit [51]. The following section presents the model fit and the interpretation. For space reasons, the class probabilities can be found in the Supporting Information (S3 Text and S3 Table). We discuss the classes just below Table 4.

The following table (Table 4) demonstrates the different goodness of fits. Model 5 with five latent classes has the lowest Bayesian information criterion (BIC), hence the best fit.

**Table 4:** Goodness of fit for latent classes corresponding to groups with different attitudes.

Model	log_likelihood	df	BIC	ABIC	CAIC	likelihood_ratio
1	-27624.46	1271	55709.52	55506.22	55773.52	14385.22
2	-26676.61	1206	54281.59	53871.82	54410.59	13645.05
3	-26098.66	1141	53593.48	52977.22	53787.48	13258.31
4	-25628.11	1076	53120.17	52297.44	53379.17	12916.53
**5**	**-25333.98**	**1011**	**52999.69**	**51970.49**	**53323.69**	**12658.13**
6	-25105.90	946	53011.32	51775.64	53400.32	12485.78

Note: BIC **=** Bayesian information criterion; ABIC = adjusted Bayesian information criterion; CAIC = consistent Akaike information criterion.

### Results of latent class analysis

The best model with five latent classes corresponds very well with a moral interpretation of WTP and especially with a classification of protest responses (class probabilities for all attributes can be found in the Supporting Information, S3 Text). The next paragraphs give a short description of each group.

Members of the first class are much more likely to be female (having a 71% probability to be female). They are the youngest group in the sample, therefore tend to have a relatively low income and have no kids (96% probability). They are concerned about animal welfare and think that animals have the same rights as humans. They are somewhat likely to be in an animal welfare organization (17%) and engage in responsible consuming (80%). Together with the third group, they have the highest probability to neither give a protest response (both definition 1 and 2) nor to be an outlier.

Members of the second class are more likely to be male (78%), are relatively young and have a lesser chance to have a university degree than other groups. This class scores much higher on the apathetic scale, is clearly less altruistic and less concerned about the environment than the other classes. There is only a 4% probability that a member of this class is in an animal welfare organization. They frequently consume meat. They are more inclined to claim a utilitarian position but at the same time possess a mix of deontological and utilitarian values. It is more probable (62%) that a member of this group thinks individuals on markets are responsible for animal welfare, not the state. Together with the fifth class they are the most likely of all five groups to give a protest response in both definitions (definition 1, protest with zero: 32%, protest only: 44%).

The third class members are older than the other classes (often 30 years +), a majority has children, a university degree and the highest income, but there are also some students in it. The probability to be female in this group is 64%–the same as in the overall sample. They frequently consume meat and often have mixed values with a slight tendency to deontological positions. In contrast to the second class, animal and human rights are both important to them, and they do consume in a responsible manner (83% probability). Together with the first group, they are the least likely of all groups to give a protest response in both definitions (protest with zero: 19% and protest only: 0%).

The fourth class is relatively homogenous in age, education and number of kids. The probability for a member to be in an animal rights group is the highest in the sample (21%). There is a 71% probability for members to be female. Animal suffering is important to them. Their moral positions are a mix of deontological and utilitarian values. They are not apathetic, and members are altruistic with the highest probability of all groups (99%). A majority thinks that animal welfare questions are the responsibility of individuals, not the state. This group can be labeled as the “outlier” group, since the probability of a member to have a WTP categorized as *outlier* (as defined above) ranges–depending on WTP question–from 61 to 83%.

Members of the final and fifth group have a probability of 69% to be female. They are likely to be students with normal income compared to the other groups. They do eat meat but less likely so than other groups. They are altruistic and are more inclined to a utilitarian value orientation. Animal rights are important to them, which corresponds well to the fact they are not apathetic. There is a relatively high chance that a member of this group is in an animal welfare organization (15%). The probability of protest bids is–like in the second group–relatively high (protest with zero: 44%, protest only 60%).

To sum up the results concerning the first research question:

- protest responses are not irrational or result from not understanding the questions.
- A substantial percentage of protesters give moral reasons for their decision.
- Latent class analysis and a statistical analysis of demographic attributes show that groups can also be clearly distinguished according to their inclination to protest.

The fourth hypothesis revolves around the relationship of zero bids and protest responses and how different definitions of what makes a protester change WTP.

*Hypothesis 4*: *WTP differs significantly with protest bids, zero bids or outliers excluded*

The following table (Table 5) provides an overview of the mean WTP for different groups described throughout the article.

**Table 5:** Mean WTP in Euro for different groups.

	Prevent killing male chicks	SD EK	More space for pigs	SD SP	Medication castration pigs	SD SK	More space for chickens	SD HP
**All**	0.97	1.41	2.89	5.68	2.61	5.73	1.03	1.49
**All without outliers**	0.58	0.51	2.10	4.91	1.72	3.95	0.65	0.68
**All without protest zero bid (Def.1)**	1.01	1.28	3.09	5.88	2.82	5.94	1.07	1.37
**All without protest only (Def. 2)**	1.02	1.41	3.01	5.64	2.78	5.77	1.04	1.23
**Outlier only**	2.81	2.43	10.63	12.68	8.31	10.24	2.94	2.43
**Protest zero bid (Def. 1)**	0.64	2.32	0.83	1.80	0.49	1.29	0.61	2.36
**Protest only** **(Def. 2)**	0.89	1.41	2.67	5.74	2.29	5.63	1.00	1.87

Note: SD = standard deviation; EK = prevent killing male chicks, SP = more space for pigs, SK = medication castration pigs, HP = more space for chickens

All differences between the normal sample and outliers, protesters (protests with zero, definition 1) or protesters (protests only, definition 2) are highly significant (Mann-Whitney-U-Test, n = 1335, p < 0.001). The two WTP for chickens for protesters (definition 2) are significant at the p < 0.01 level. Hence, hypothesis 5 is supported. Note that WTP for protesters (protests with zero, definition 1) is not zero, because the inclusion criterion for this group is a zero bid in either one of the four WTP questions, not necessarily all of them. Note also that the outlier group is rather big, as 298 out of 1335 participants (22%) state a WTP in all four animal welfare questions that is 1.5 times the interquartile range.

A follow-up question to this hypothesis is whether a more inclusive definition of protest bids (protests only, definition 2) leads to higher instead of lower WTP than normal bids. Table 5 shows that protesters with a zero WTP and protest response (definition 1) are consistently below the first group (only protest, definition 2) in respect to their WTP. Both protest groups always show a lower WTP than the rest of the sample.

## Discussion

The first goal of this article was to investigate motivations behind protest responses for WTP for animal welfare. It has become clear that only a marginal part (3%) does not answer at all, 4% need more information and another 4% guessed or invented WTP. This is in line with other surveys and supports the conclusion that protest bids are not due to irrationality, lack of understanding or a refusal to answer. On the contrary, a higher percentage of protest bids need more information (10–15%) and many participants feel the necessity to explain their reasons in the comments.

Measuring protest responses in two different ways enabled us to find out whether protest bids necessarily have to be associated with a zero WTP. A first finding, replicating other studies [12,17], shows protest responses to exist independently from zero WTP statements–in our sample 22% of all protest responses are not associated with a zero WTP, whereas 8% are.

**Fig 1:**
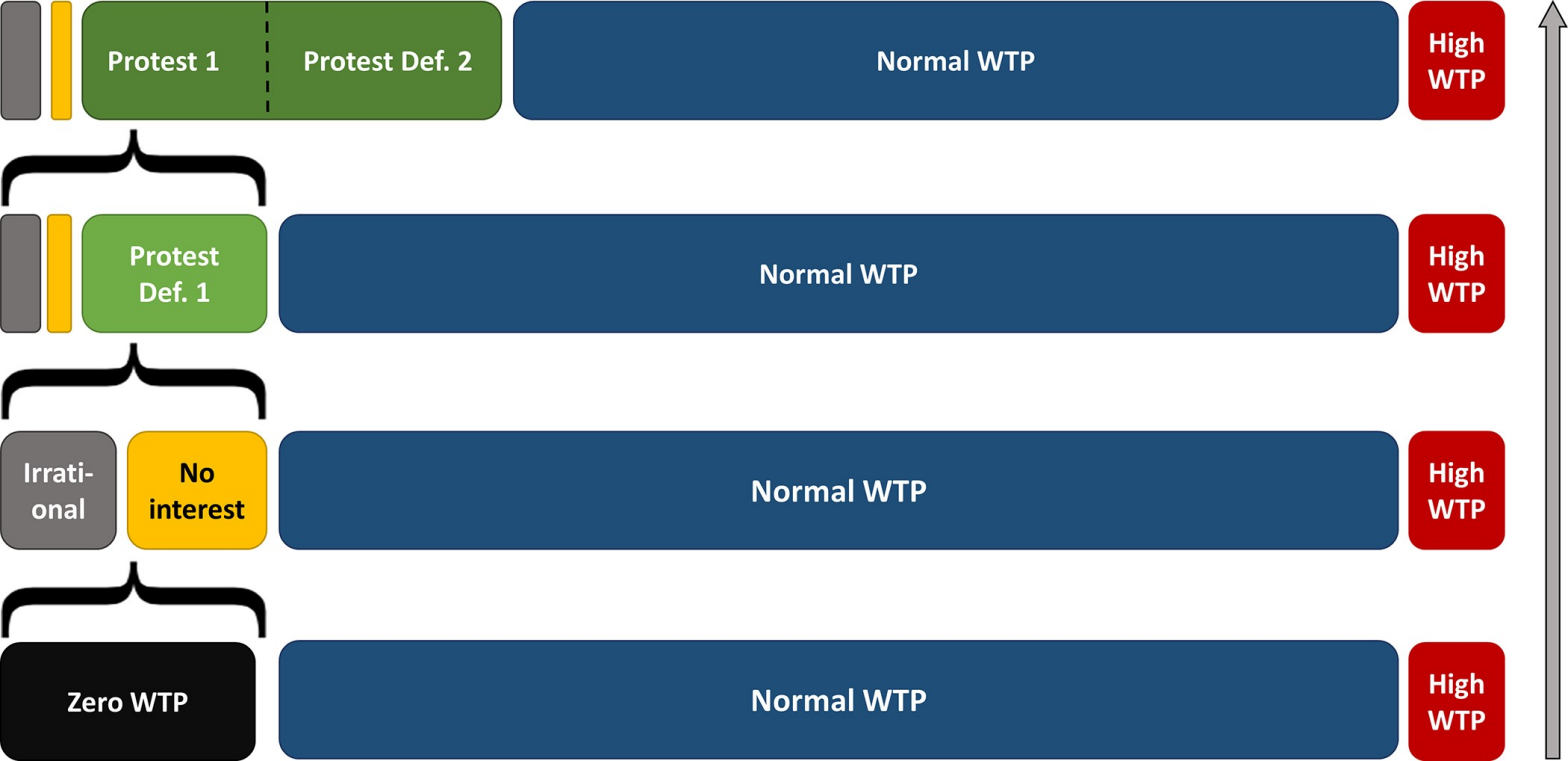
Conceptualization of WTP-answers.

These results lead us to a new way to conceptualize WTP answers–depicted in Fig 1.

The figure has to be read from bottom to top with each bar representing 100% of a WTP sample. The ascending order depicts different conceptualizations and differentiations of subjects with a zero WTP. To begin, the bottom bar illustrates the traditional way to separate groups–there are zero WTP participants, a majority with a “normal” WTP and some outliers. Often, both zero bids and outliers are censored, i.e. removed from the sample. However, if motivations are explored further, the zero WTP group can be split in those with no interest for the issue in question (i.e. a genuine WTP of zero) and those with irrational answers. Yet, only a small fraction of this group is truly irrational (this study: 3%) or can be classified as true zero answers (this study: 1–2%). The majority of this group, in turn, shows a zero WTP for other reasons. Furthermore, the top bar demonstrates that the group of protest bids may even extend into the group of normal WTP if protesters are not conceived as having necessarily a WTP of zero [12,16,13].

It seems also possible to differentiate protest responses in terms of demographics and attitudes. For both protest definitions, differences in gender, age, education and income were significant. Protesters, on average, are younger, less educated, have a lower income and fewer kin to care for (Table 3). There are little or no differences, however, concerning intrinsic values of animals or the attitude towards utilitarian values.

Most notably, a latent class analysis allows to clearly distinguishing protesters by identifying two groups with a “normal” WTP (a younger group (group 1) and an older one (group 3)). Two of the remaining groups have an increased inclination to protest and there is one group of outliers. The outliers particularly are distinguishable in their characteristics, since they are very much concerned about animal welfare, are highly altruistic and not apathetic at all.

Since some studies treat protest responses as a true WTP and calculate accordingly, our study investigated whether this procedure is acceptable. For animal welfare, it clearly does not seem to be the case, as only 0.4% of all zero bids (definition 1) state that animal welfare is no goal for them. On the contrary, the great majority of protesters express strong pro-animal welfare opinions. Therefore, this study recommends differentiating between true zero bids which should be only a few, depending on topic, and protest zero bids.

In addition, it has been a long-standing question whether moral attitudes are important for protest responses [12,8]. Our sample confirms that many protest responses attribute their protest directly to moral reasons.

Finally, there has been much debate whether the estimation of WTP is biased by including or excluding the protest responses [30,17]. Our study shows in great detail (Table 5) that, indeed, WTP estimations are highly significantly different depending on the inclusion or exclusion of protest responses or outliers according to our two definitions of protest responses. Excluding protest responses leads to a consistently higher WTP, excluding outliers changes the estimates the most. In any case, excluding either group leads to significantly different estimations of WTP for animal welfare (Table 5).

At this point, we want to stress the treatment of outliers who form a sizeable group in our sample (22%) and have an immense impact on WTP. Excluding them almost halves the willingness to pay for animal welfare aspects. If analyzed separately, outliers are, on average, prepared to pay about 3–4 times the amount of the good in question! It has also been suggested that protest responses do not necessarily imply an attitude of no value for the good in question but a dissatisfaction with the way the issue in question is framed by the survey [30]. Our study does not support this conclusion but stresses the moral attitude behind WTP decisions.

The latent class analysis lends some support for a protesters profile (younger, low income, less educated) that agrees with profiles of other studies [6,31,12]. However, we can assume that protest and WTP decisions are highly problem-specific. One cue may be that other studies find other reasons given for WTP, e.g. in one study about biodiversity and water supply risks 27% stated that they cannot afford it [26]. In our study, this answer practically does not show up in the large body of free text entries. Another cue comes from just these text entries which provide a wealth of contextual information. Very many entries converge on a highly specific argument, namely that they disagree with paying for any single aspect of improving animal welfare, but are in strong favor of paying for an all-around guarantee that animals are treated well.

Thus, these participants seem to disagree with an individual market-approach of paying for single animal welfare aspects. On the other hand, they state that they would pay high sums (around 10 Euro) if there were a "complete package" ensuring animal welfare by providing everything in a manner that is appropriate for the species. In the survey, these individuals do not appear as outliers but state a "normal" WTP. However, by framing the questions differently they almost certainly would have emerged as exactly that.

## Conclusion

The percentage of protesters in contingent valuation surveys is very likely substantial–a meta-study estimates its share at around 18% [15]. A usually substantial group of outliers add to this methodological problem, since it is largely unclear whether the estimation of WTP is more biased by the exclusion or inclusion of protest bids. Proper treatment is difficult, because motivations behind protest responses are largely unclear, definitions of protest differ between studies and often only those survey participants that state a zero willingness to pay are asked for their reasons.

This paper seeks to clarify the motivations behind protest answers by providing a synthesis of possible reasons of the literature for a particularly suited topic–farm animal welfare–to all survey participants. A further distinction between two possible protest definitions and an outlier group demonstrates the sensitivity of WTP estimates concerning inclusion or exclusion of these groups. These two definitions follow two theoretical approaches; the first regards WTP as economic preference [20], the other one as result from certain attitudes [12,13]. The latter view implies that zero bids may be an expression of protest based on moral and other values. Hence, according to this regard, contingent valuation should be reconsidered as an economic tool [7].

Our findings confirm that protest bids are not irrational, driven by lack of understanding or simply a refusal to answer. Moreover, a large part is directly motivated by moral reasons. Protesting individuals for animal welfare issues are significantly different from the other participants as shown by the latent class analysis.

Furthermore, protest responses are not necessarily coupled to a zero WTP. On the contrary, 8% out of 32% protesting individuals have a zero WTP. This finding throws a critical light on the usual definition of protest. At the same time, only a small fraction of zero bids (0.4%) are true WTP statements, i.e. respondents were satisfied with the status quo, so that further animal welfare improvements are no goal for them. This very small fraction means in practice that protest bids should not be treated as true zero bids, as is sometimes the case.

Finally, we could show that WTP estimates are indeed significantly biased by the exclusion of protesters. Excluding protesters according to a strict definition leads to a higher WTP in the remaining sample.

These differences lead us to conclude that motivations behind willingness to pay, the topic involved and the definition of protest in contingent valuation studies is highly sensitive to small variations. Therefore, in our opinion, it is highly important to converge on comparable definitions of protest responses, to apply the same treatment to protesters and to be careful with generalizations across environmental topics.

## Supporting information

S1 Text Wording for WTP-questions. (DOC)

S2 Text Description of scales. (DOC)

S3 Text Classes and results of latent class analysis. (DOC)

S1 Table Factor loadings for GAC-scale (two factors). (DOC)

S2 Table Factor loadings for GAC-scale (three factors). (DOC)

S3 Table LCA-analysis: Binning of variables. (DOC)
