# Supplementary material for: Distinguishing protest responses in contingent valuation: A conceptualization of motivations and attitudes behind them
Source: PLoS One. 2019 Jan 8;14(1):e0209872. doi: 10.1371/journal.pone.0209872 (PMC6324805; doi:10.1371/journal.pone.0209872)
Supplement: S1 Text — (DOC) [file pone.0209872.s001.doc]

# Wording for WTP-questions

1. To produce eggs only female animals are needed. Male chickens are therefore killed on the first day for economic reasons. At the moment six eggs from deep litter farming cost 1.32 Euro. How much more would you pay for six eggs if male chickens could be raised as mast animals (in Eurocent)?
2. In accordance with the German animal protection law fattened pigs are allowed to have, depending on their weight, between 0.5 and 1 m² space. Animal rights activists demand more space. At the moment, a chop of meat from pigs (1 kg) costs around 4.95 Euro. How much more would you pay for 1 kg of pork if pigs would get 1 m² more space (in Eurocent)?
3. Uncastrated male fattened pigs may develop a boar smell which may be tasted in the pork. Such pork is unsellable. Therefore male piglets may be castrated during the first seven days after birth without anesthesia. Castration with anesthesia is more expensive and therefore is often skipped for economic reasons. At the moment, a chop of meat from pigs (1 kg) costs around 4.95 Euro. How much more would you pay for 1 kg of pork if male piglets would get anesthesia when castrated (in Eurocent)?
4. In accordance with the German animal protection law laying hens are allowed to have a space of 27 x 30 cm, which is a bit less than 1 1 /2 DIN A4 sheets. Animal rights activists demand more space. At the moment six eggs from deep litter farming cost 1.32 Euro. How much more would you pay for six eggs if each laying hen would get around 300 cm² more space (in Eurocent)?
