# Supplementary material for: Distinguishing protest responses in contingent valuation: A conceptualization of motivations and attitudes behind them
Source: PLoS One. 2019 Jan 8;14(1):e0209872. doi: 10.1371/journal.pone.0209872 (PMC6324805; doi:10.1371/journal.pone.0209872)
Supplement: S2 Text — (DOC) [file pone.0209872.s002.doc]

# Description of scales

## GAC-scale

The scale used for environmental concern is called GAC scale [5,6] and has been widely used. The nine questions are as follows and could be answered on a 1-5 point-scale ranging from "Totally disagree" to "Totally agree". We constructed an index out of these answers, which is simply the sum of all values, therefore ranging from 5 to 45.

1. Environmental protection benefits everyone.

2. Over the next decade, thousands of species of plants and animals will become extinct.

3. Claims that we are changing the climate are greatly exaggerated.

4. While some local plants and animals may have been harmed by environmental degradation, over the whole Earth there has been little effect.

5. Environmental threats to public health have been exaggerated.

6. Environmental protection is beneficial to my health.

7. Environmental protection will provide a better world for me and my children.

8. Environmental protection will help me to have a better quality of life.

9. Environmental damage generated here harms people all over the world.

## Altruistic value orientation

The four items are taken from the „Helping attitude scale (HAS)“ [2] and could be answered on a 1-5 point scale ranging from "Totally disagree" to "Totally agree". We constructed an index out of these answers, which is simply the sum of all values, therefore ranging from 4 to 20.

1. If a person in front of me at the supermarket checkout has a few cents too little, I pay the difference.

2. I feel good when I can help someone else.

3. It is an important goal of education to teach children to help others.

4. I help others even if they are strangers to me.

## Apathic value orientation

This scale is adapted from [1] and could be answered on a 1-5 point scale ranging from "Totally disagree" to "Totally agree". We constructed an index out of these answers, which is simply the sum of all values, therefore ranging from 3 to 15.

1. Environmental threats such as deforestation and climate warming have been exaggerated.

2. Given enough time, most environmental problems will solve themselves.

3. Too much emphasis has been placed on conservation.

## Deontological / utilitarian value orientation

The questions constitute the validated Robinson scale [4]. Questions 1, 3, 5, 7, 9 constitute the deontological, questions 2, 4, 6, 8, 10 the utilitarian value orientation. The 10 questions could be answered on a 1-5 point scale ranging from "Totally disagree" to "Totally agree". We constructed an index out of these answers, which is simply the sum of all values, therefore ranging from 10 to 50.

What is your opinion about the following statements:

1. It is never morally justified to cause someone harm.
2. Rules and laws are irrelevant; whether an action produces happiness is all that matters when deciding how to act.
3. If an action is a violation of society’s most basic rules it should not be committed; even if it will result in a large amount of good.
4. Rules and laws should only be followed when they maximize happiness.
5. Some aspects of humanity are sacred and should never be violated no matter the possible gain.
6. If rules and laws do not maximize happiness for people they should be ignored.
7. Some rules and laws are universal and are binding no matter the circumstances you find yourself in.
8. The only moral principle that needs to be followed is that one must maximize happiness.
9. Some rules should never be broken.
10. People that fail to maximize happiness are doing something morally wrong.

# References

1. Gagnon Thompson SC, Barton MA (1994) Ecocentric and anthropocentric attitudes toward the environment. J Environ Psychol 14 (2): 149–157.

2. Nickell GS (1998) The Helping Attitude Scale: A new measure of prosocial tendencies. Available online at: http://web.mnstate.edu/nickell/HAS20%20Plus%20Scoring%20and%20Citations.doc

4. Robinson JS (2012) The Consequentialist Scale: Elucidating the Role of Deontological and Utilitarian Beliefs in Moral Judgments. Available online at: http://hdl.handle.net/1807/33868

5. Stern PC, Dietz T, Guagnano GA (1995) The New Ecological Paradigm in Social-Psychological Context. Environ Behav 2 7(6): 723–743.

6. Stern PC, Dietz, T, Kalof L (1993) Value Orientations, Gender, and Environmental Concern. Environ Behav 25 (5): 322–348.
