# Supplementary material for: Distinguishing protest responses in contingent valuation: A conceptualization of motivations and attitudes behind them
Source: PLoS One. 2019 Jan 8;14(1):e0209872. doi: 10.1371/journal.pone.0209872 (PMC6324805; doi:10.1371/journal.pone.0209872)
Supplement: S3 Text — (DOC) [file pone.0209872.s003.doc]

# Classes and results of latent class analysis

Conditional item response (column) probabilities, by outcome variable, for each class (row), calculated with R 3.2.3, package poLCA [3]. The binning of variables is explained below.

Age

Pr(1) Pr(2) Pr(3) Pr(4) Pr(5) Pr(6)

class 1: 0.1198 0.8166 0.0330 0.0000 0.0000 0.0306

class 2: 0.0949 0.5681 0.1068 0.0692 0.1157 0.0454

class 3: 0.0000 0.1406 0.3466 0.2024 0.2766 0.0337

class 4: 0.0432 0.4239 0.2356 0.1083 0.1584 0.0307

class 5: 0.0808 0.5199 0.1408 0.0984 0.0993 0.0608

Sex

Pr(1) Pr(2)

class 1: 0.7075 0.2925

class 2: 0.2193 0.7807

class 3: 0.6359 0.3641

class 4: 0.7052 0.2948

class 5: 0.6936 0.3064

Income

Pr(1) Pr(2) Pr(3) Pr(4) Pr(5) Pr(6)

class 1: 0.3163 0.4305 0.1475 0.0579 0.0277 0.0200

class 2: 0.1580 0.3354 0.1028 0.0856 0.1218 0.1964

class 3: 0.0000 0.0503 0.2085 0.2093 0.2156 0.3163

class 4: 0.1180 0.3033 0.1347 0.1279 0.1314 0.1847

class 5: 0.2420 0.3539 0.1421 0.1778 0.0078 0.0764

Educational level

Pr(1) Pr(2) Pr(3) Pr(4)

class 1: 0.3024 0.0683 0.6293 0.0000

class 2: 0.2204 0.0211 0.7585 0.0000

class 3: 0.0133 0.1740 0.8049 0.0078

class 4: 0.1339 0.1053 0.7608 0.0000

class 5: 0.2025 0.1168 0.6807 0.0000

Responsibility for kin

Pr(1) Pr(2) Pr(3) Pr(4)

class 1: 0.9630 0.0370 0.0000 0.0000

class 2: 0.7447 0.0664 0.0949 0.0940

class 3: 0.3716 0.3210 0.1998 0.1075

class 4: 0.7109 0.1363 0.0946 0.0583

class 5: 0.7671 0.1377 0.0765 0.0187

Role of animal welfare in legislation

Pr(1) Pr(2) Pr(3) Pr(4) Pr(5)

class 1: 0.2653 0.5226 0.1550 0.0452 0.0120

class 2: 0.0000 0.1809 0.5690 0.1883 0.0617

class 3: 0.2821 0.5212 0.1558 0.0274 0.0134

class 4: 0.4264 0.3584 0.1124 0.0903 0.0124

class 5: 0.3101 0.4035 0.2470 0.0393 0.0000

Role of animal welfare in comparison to human welfare

Pr(1) Pr(2) Pr(3) Pr(4) Pr(5)

class 1: 0.0369 0.0467 0.5429 0.2941 0.0794

class 2: 0.0000 0.0122 0.0504 0.2543 0.6830

class 3: 0.0161 0.0248 0.5313 0.2748 0.1531

class 4: 0.0185 0.0123 0.6075 0.2510 0.1106

class 5: 0.0387 0.0078 0.5225 0.3506 0.0804

Responsible consumer behavior

Pr(1) Pr(2) Pr(3)

class 1: 0.7994 0.1254 0.0752

class 2: 0.3259 0.6055 0.0686

class 3: 0.8266 0.0962 0.0772

class 4: 0.9260 0.0500 0.0239

class 5: 0.7878 0.1343 0.0779

Absolute rights of humans

Pr(1) Pr(2) Pr(3)

class 1: 0.8653 0.0717 0.0629

class 2: 0.8733 0.1188 0.0079

class 3: 0.8968 0.0688 0.0344

class 4: 0.8593 0.0776 0.0630

class 5: 0.8809 0.0687 0.0505

Absolute rights of animals

Pr(1) Pr(2) Pr(3)

class 1: 0.7902 0.0955 0.1144

class 2: 0.2321 0.5815 0.1864

class 3: 0.7704 0.1218 0.1077

class 4: 0.7328 0.1030 0.1642

class 5: 0.7064 0.1281 0.1654

Knowledge on animal welfare

Pr(1) Pr(2) Pr(3) Pr(4) Pr(5)

class 1: 0.0042 0.1116 0.4425 0.3787 0.0629

class 2: 0.0535 0.2940 0.2977 0.2621 0.0927

class 3: 0.0134 0.1453 0.4185 0.3498 0.0730

class 4: 0.0060 0.0862 0.3339 0.4197 0.1542

class 5: 0.0256 0.0899 0.4099 0.3453 0.1293

Animal welfare as moral issue

Pr(1) Pr(2) Pr(3) Pr(4) Pr(5)

class 1: 0.0436 0.2145 0.4043 0.2579 0.0797

class 2: 0.2665 0.3836 0.2101 0.1085 0.0314

class 3: 0.0728 0.2677 0.3349 0.2599 0.0647

class 4: 0.0807 0.2067 0.2683 0.2706 0.1736

class 5: 0.0547 0.2317 0.3803 0.1855 0.1478

Member of an animal welfare organisation

Pr(1) Pr(2)

class 1: 0.1665 0.8335

class 2: 0.0444 0.9556

class 3: 0.1746 0.8254

class 4: 0.2053 0.7947

class 5: 0.1532 0.8468

Deontological/utilitarian attitude 1

Pr(1) Pr(2) Pr(3)

class 1: 0.7176 0.0770 0.2054

class 2: 0.5035 0.2529 0.2436

class 3: 0.7523 0.0547 0.1930

class 4: 0.7770 0.0832 0.1397

class 5: 0.6533 0.1221 0.2246

Deontological/utilitarian attitude 2

Pr(1) Pr(2)

class 1: 0.4356 0.5644

class 2: 0.3755 0.6245

class 3: 0.4003 0.5997

class 4: 0.4464 0.5536

class 5: 0.4617 0.5383

Deontological/utilitarian attitude 3

Pr(1) Pr(2)

class 1: 0.3939 0.6061

class 2: 0.4942 0.5058

class 3: 0.5578 0.4422

class 4: 0.4888 0.5112

class 5: 0.4114 0.5886

Apathy

Pr(1) Pr(2)

class 1: 0.0064 0.9936

class 2: 0.3296 0.6704

class 3: 0.0517 0.9483

class 4: 0.0814 0.9186

class 5: 0.0458 0.9542

Apathy towards environmental concerns

Pr(1) Pr(2)

class 1: 0.0434 0.9566

class 2: 0.3032 0.6968

class 3: 0.0691 0.9309

class 4: 0.0447 0.9553

class 5: 0.0431 0.9569

Altruism

Pr(1) Pr(2)

class 1: 0.9593 0.0407

class 2: 0.8607 0.1393

class 3: 0.9667 0.0333

class 4: 0.9895 0.0105

class 5: 0.9518 0.0482

Animal welfare as individual responsibility

Pr(1) Pr(2)

class 1: 0.3765 0.6235

class 2: 0.6897 0.3103

class 3: 0.4056 0.5944

class 4: 0.3484 0.6516

class 5: 0.4692 0.5308

Frequency of meat consumption

Pr(1) Pr(2) Pr(3)

class 1: 0.7151 0.2829 0.0021

class 2: 0.9504 0.0496 0.0000

class 3: 0.8329 0.1671 0.0000

class 4: 0.5738 0.4262 0.0000

class 5: 0.5762 0.4110 0.0128

Protest definition 1 (zero bid + debriefing)

Pr(1) Pr(2)

class 1: 0.0000 1.0000

class 2: 0.3198 0.6802

class 3: 0.0000 1.0000

class 4: 0.0000 1.0000

class 5: 0.4412 0.5588

Protest definition 2 (debriefing only)

Pr(1) Pr(2)

class 1: 0.3099 0.6901

class 2: 0.5196 0.4804

class 3: 0.1927 0.8073

class 4: 0.2305 0.7695

class 5: 0.5983 0.4017

WTP for not killing male chicks

Pr(1) Pr(2) Pr(3) Pr(4)

class 1: 0.0192 0.8769 0.1006 0.0033

class 2: 0.3450 0.6295 0.0255 0.0000

class 3: 0.0080 0.9210 0.0709 0.0000

class 4: 0.0000 0.1669 0.8331 0.0000

class 5: 0.2315 0.5489 0.0832 0.1364

WTP for more space for pigs

Pr(1) Pr(2) Pr(3) Pr(4)

class 1: 0.0043 0.9957 0.0000 0.0000

class 2: 0.2877 0.6969 0.0085 0.0068

class 3: 0.0000 0.9927 0.0073 0.0000

class 4: 0.0000 0.3844 0.6156 0.0000

class 5: 0.5216 0.2515 0.0169 0.2101

WTP for pain relief for castration of pigs

Pr(1) Pr(2) Pr(3) Pr(4)

class 1: 0.0077 0.9538 0.0349 0.0036

class 2: 0.3917 0.5776 0.0002 0.0305

class 3: 0.0083 0.9544 0.0297 0.0075

class 4: 0.0000 0.2266 0.7734 0.0000

class 5: 0.5852 0.1858 0.0067 0.2223

WTP for more space for chickens

Pr(1) Pr(2) Pr(3) Pr(4)

class 1: 0.0146 0.9521 0.0237 0.0096

class 2: 0.3293 0.6451 0.0121 0.0135

class 3: 0.0053 0.9802 0.0130 0.0015

class 4: 0.0066 0.2970 0.6785 0.0179

class 5: 0.3169 0.4351 0.0452 0.2028

**Estimated class population shares**

Class 1: 35.77

Class 2: 10.94

Class 3: 28.96

Class 4: 12.56

Class 5: 11.77

**Predicted class memberships (by modal posterior prob.)**

Class 1: 37%

Class 2: 11%

Class 3: 28%

Class 4: 13%

Class 5: 11%

=========================================================

Fit for 5 latent classes:

=========================================================

number of observations: 1335

number of fully observed cases: 468

number of estimated parameters: 324

residual degrees of freedom: 1011

maximum log-likelihood: -25333.98

AIC(5): 51315.97

BIC(5): 52999.69

G^2(5): 12658.13 (Likelihood ratio/deviance statistic)

X^2(5): 1.323362e+12 (Chi-square goodness of fit

# References

3. R Core Team (2016) R: A Language and Environment for Statistical Computing. Available online at: https://www.R-project.org/
