# Supplementary material for: Distinguishing protest responses in contingent valuation: A conceptualization of motivations and attitudes behind them
Source: PLoS One. 2019 Jan 8;14(1):e0209872. doi: 10.1371/journal.pone.0209872 (PMC6324805; doi:10.1371/journal.pone.0209872)
Supplement: S2 Table — (DOC) [file pone.0209872.s005.doc]

## Factor loadings GAC

Factor analysis was performed with R 3.3.1, package stats [3]. The factor loadings for the GAC scale with three factors and a varimax rotation are as follows:

**Table 1**

Factor loadings for the GAC-scale (two factors, varimax rotation)

| Factor 1 | Factor 2 | Uniqueness |
| --- | --- | --- |
| 0.455 | 0.285 | 0.711 |
| 0.207 | 0.305 | 0.864 |
| 0.171 | 0.624 | 0.581 |
| 0.158 | 0.688 | 0.502 |
| 0.258 | 0.638 | 0.526 |
| 0.778 | 0.220 | 0.346 |
| 0.849 | 0.216 | 0.232 |
| 0.828 | 0.226 | 0.263 |
| 0.442 | 0.350 | 0.682 |

The corresponding Chi^2^-statistic is χ2 = 82.14, df = 19, p < 0.001.

# References

3. R Core Team (2016) R: A Language and Environment for Statistical Computing. Available online at: https://www.R-project.org/
