# Supplementary material for: Distinguishing protest responses in contingent valuation: A conceptualization of motivations and attitudes behind them
Source: PLoS One. 2019 Jan 8;14(1):e0209872. doi: 10.1371/journal.pone.0209872 (PMC6324805; doi:10.1371/journal.pone.0209872)
Supplement: S3 Table — (DOC) [file pone.0209872.s006.doc]

# LCA-analysis: Binning of variables

**Table 4**

LCA-analysis: Binning of variables

|  | **Pr(1)** | **Pr(2)** | **Pr(3)** | **Pr(4)** | **Pr(5)** | **Pr(6)** |
| --- | --- | --- | --- | --- | --- | --- |
| **Age** | < 21 years | 21-30 | 31-40 | 41-50 | 51-60 | > 60 |
| **Sex** | Female | Male |  |  |  |  |
| **Income** | < 500 Euro | 500-1500 | 1500-2500 | 2500-3500 | 3500-4500 | > 4500 |
| **Educational level** | 1-3 | 4-6 | >= 7 | NA |  |  |
| **Responsibility for kin** | 1 | 2 | 3 | >= 4 | NA |  |
| **Role of animal welfare in legislation** | Very important | Important | Neither important nor unimportant | Unimportant | Very unimportant |  |
| **Role of animal welfare in comparison to human welfare** | AW much higher than human welfare | AW somewhat higher than human welfare | Both are equally important | Human welfare somewhat higher than animal welfare | Human welfare much higher than animal welfare |  |
| **Responsible consumer behavior** | Yes | No | Don't know |  |  |  |
| **Absolute rights of humans** | Yes | No | Don't know |  |  |  |
| **Absolute rights of animals** | Yes | No | Don't know |  |  |  |
| **Knowledge on animal welfare** | Very low | Low | Neither low nor high | High | Very High |  |
| **Animal welfare as moral issue** | Totally disagree | Disagree | Neither disagree nor agree | Agree | Totally agree |  |
| **Member of an animal welfare organisation** | Yes | No |  |  |  |  |
| **Deontological/utilitarian attitude 1** | More of a mix | No mix | Don't know |  |  |  |
| **Deontological/utilitarian attitude 2** | More deontological | More utilitarian |  |  |  |  |
| **Deontological/utilitarian attitude 3** | More deontological | More utilitarian |  |  |  |  |
| **Apathy** | More apathetic | Less apathetic |  |  |  |  |
| **Apathy towards environmental concerns** | More apathetic | Less apathetic |  |  |  |  |
| **Altruism** | More altruistic | Less altruistic |  |  |  |  |
| **Animal welfare as individual responsibility** | Yes | No |  |  |  |  |
| **Frequency of meat consumption** | Frequently | Rarely | Never |  |  |  |
| **Protest definition 1 (zero bid + debriefing)** | Yes | No |  |  |  |  |
| **Protest definition 2 (debriefing only)** | Yes | No |  |  |  |  |
| **WTP for not killing male chicks** | 0 | > 0 & < 198 | > 198 | NA |  |  |
| **WTP for more space for pigs** | 0 | > 0 & < 736.5 | > 736.5 | NA |  |  |
| **WTP for pain relief for castration of pigs** | 0 | > 0 & < 589.5 | > 589.5 | NA |  |  |
| **WTP for more space for chickens** | 0 | > 0 & < 217.5 | > 217.5 | NA |  |  |
